# Prevalence of HIV and sexually transmitted infections among young women engaged in sex work aboard foreign fishing vessels in Kiribati

**DOI:** 10.5365/wpsar.2017.8.4.005

**Published:** 2018-03-23

**Authors:** Tebuka Toatu, Paul White, Damian Hoy, Dennie Iniakwala, Onofre Edwin A Merrilles, Sameer Vali Gopalani

**Affiliations:** a Public Health Division, Pacific Community, Noumea, New Caledonia.; b Epidemiology and Laboratory Capacity Program, Public Health and Hospital Emergency Preparedness Office, Commonwealth Health Care Corporation, Saipan, Commonwealth of the Northern Mariana Islands.; c Department of Biostatistics and Epidemiology, College of Public Health, University of Oklahoma Health Sciences Center, Oklahoma City, OK, USA.

## Abstract

**Objective:**

To assess the prevalence of HIV and sexually transmitted infections (STIs) among women who board foreign fishing vessels for sex work in Kiribati.

**Methods:**

A cross-sectional study was designed to collect data on behavioural risk factors for STIs and knowledge of and attitudes towards HIV and STIs during 2007. Urine and blood samples were collected to test for HIV and select STIs. Descriptive statistics were performed for sociodemographic and behavioural characteristics, and χ^2^ tests were used to assess associations between potential key determinants and the presence of genital *Chlamydia* infection.

**Results:**

Women who boarded foreign fishing vessels for transactional sex were younger, had less education, were less likely to live with a partner and were less likely to be otherwise employed. Although no HIV infections were detected, more than half (57.5%) of the women were diagnosed with an STI. One quarter of the women tested positive for chlamydia, and 40% tested positive for mycoplasma. The presence of chlamydia was strongly associated with age at first sexual intercourse (*P* = 0.02) and number of sexual partners during the prior seven days (*P* = 0.02).

**Conclusion:**

The high rate of STIs in this population of sex workers is concerning given the potential of severe pregnancy-related and chronic health problems and the increased risk of transmission within the general population of Kiribati. We identified a specific sex worker population as a priority group for targeted public health efforts to prevent and control the spread of STIs in Kiribati.

## Introduction

Kiribati is a small island developing state composed of 33 widely dispersed small islands in the northern Pacific Ocean. The country has a population of 92 533, of whom 57.9% are under 25 years of age. (*1*) Kiribati’s large exclusive economic zone, which covers 3.5 million kilometres of ocean, is an important national economic resource, generating income from fishing licenses, access fees and transhipment fees from foreign fleets. The income gained from foreign fishing vessels is critical to local and national economies. Despite the economic benefits, unfavourable consequences have arisen such as an expansion of transactional sex (defined as sex in exchange for money or resources) in Kiribati among fishermen and seafarers from Asia and other Pacific countries with Kiribati women.

Transactional sex in Kiribati is on the rise. The estimated number of women engaging in transactional sex on relatively urban South Tarawa doubled from 30–50 in 2003 to 60–100 in 2006. (*2*) The increasing trend is also apparent on Kiritimati island. (*2*)

Foreign fishing vessel crews are in port from days to weeks, during which time local women board the vessels for transactional sex. Some of these women drink alcohol with the foreign fishermen and also engage in sex with local men. Excessive alcohol consumption is prevalent among both men and women, which may lead to sexual assault. Also, the frequency of condom use during these encounters is low. (*2*)

Kiribati has an estimated 55 cumulative cases of human immunodeficiency virus (HIV) dating from 1991, (*3*) and the incidence rate for HIV is among the highest in the Pacific region. (*4*, *5*) In Kiribati, the most frequently reported mode of HIV transmission is heterosexual contact followed by perinatal transmission. (*3*) In a meta-analysis of studies from 50 low- and middle-income countries, the HIV prevalence among female sex workers was 11.8%. (*6*) In the Pacific, a meta-analysis reported a sexually transmitted infection (STI) prevalence of 28.7% in Papua New Guinea. (*7*) In Kiribati, the total STI incidence reported from 2000 to 2005 was 30.1 per 10 000 people. Most (62.9%) of the cases were males. However, these figures mask the true burden due to underreporting as STI reporting in Kiribati is limited by laboratory and public health capacity. (*8*)

At the national level, surveillance and reporting of HIV/AIDS has been in place since 1989. HIV cases are reported by sentinel laboratories to the HIV Programme Coordinator. The Coordinator prepares monthly HIV reports with information on sex, age and mode of transmission. At the time of the study, surveillance and reporting for other STIs were limited by the lack of laboratory and public health information capacity. Besides HIV, other notifiable STIs were gonorrhoea and syphilis. Chlamydia was not a notifiable infection, and testing facilities were not available locally during the time of this study.

In a combined HIV surveillance survey and behavioural surveillance survey from 2004 to 2005, as part of the Second Generation Surveillance Surveys of HIV, other STIs and Risk Behaviours in 6 Pacific Island Countries, seafarers were identified as a high-risk group for both HIV and STIs. (*9*) Overall, 9.3% of the seafarers were infected with genital *Chlamydia trachomatis*. (*9*) In a report specific to Kiribati, women involved in transactional sex were identified to be at high risk for STIs. (*3*) Women who board foreign fishing vessels for sex are thought to be at particular high risk since they often have multiple partners on the fishing vessels and within the local community.

Although two studies on behavioural risk factors among youth and young women boarding foreign fishing vessels are available, (*10*, *11*) neither of these studies includes information on the prevalence for STIs and HIV in this population. To address this important gap, the present study was undertaken to determine the prevalence of HIV and STIs and assess behavioural risk factors among women engaged in transactional sex on foreign fishing vessels in Kiribati.

## Methods

### Study design

A cross-sectional study was undertaken to determine the HIV and STI prevalence and assess behavioural risk factors among women engaged in transactional sex with foreign seafarers.

### Setting

The study was undertaken at two sites in Kiribati: South Tarawa from January to March 2007 and Kiritimati island from May to June 2007. It was structured in the form of a three-day workshop, with days one and two dedicated to participant interviews and sample collection and day three reserved for providing education on STIs and HIV, access to and use of contraceptives, voluntary counselling, confidential testing and human rights.

### Participants

As transactional sex contravenes Kiribati custom, a multisectoral approach was adopted to identify women engaged in sex work with foreign seafarers. We worked in conjunction with government agencies, nongovernmental organizations and trained peer educators from the Adolescent Health Development centre to identify, inform and invite potential participants into the study in person. Due to the absence of trained peer educators on Kiritimati, trained counsellors were used. Women who were 18–33 years old, resided in South Tarawa or Kiritimati island and boarded a foreign fishing vessel for sex work within the previous 12 months were invited to participate in the study. The age cut-offs were selected because women below 18 years were unable to provide consent for the study and those above 33 years were unlikely to be involved in transactional sex on foreign fishing vessels. Of the 83 women invited to participate in the study, 80 women met the eligibility criteria. All eligible women agreed to participate in the study. Prior to enrolment, information on the study was provided verbally and through an information sheet. Participation was voluntary, and a signed consent form was obtained from all participants. All participants were compensated with an amount of 10 Australian dollars. Any women found to have STIs were managed in accordance to the World Health Organization Sexually Transmitted Infections Case Management Guidelines as adapted by the Ministry of Health and Medical Services in Kiribati. Additionally, pre- (before sample collection) and post-test (after receipt of test results) counselling was provided by nurses and officers who had undergone counselling training.

### Sample size

Based on the prevalence of chlamydia at 20% among women under 20 years attending an antenatal clinic in Tarawa (2002–2003), (*9*) we calculated a target sample size of 88. Sample size was calculated using PS: Power and Sample Size Calculation, version 2.1.31 (William D. Dupont and W. Dale Plummer, Jr., Memphis, Tennessee, USA).

### Data and specimen collection

We developed a structured cross-sectional questionnaire and collected blood and urine samples. The questionnaire comprised 65 questions across 11 sections (see Annex 1), and it was also translated from English to Kiribati language. First, trained counsellors administered one-on-one interviews using the questionnaire with quantitative and qualitative items. During each interview, which lasted 20–30 minutes per participant, counsellors collected information on sociodemographic characteristics, behavioural risk factors for STIs and knowledge of and attitudes towards STIs and HIV. Second, urine and blood samples were collected. Serological testing of blood samples for HIV, syphilis and hepatitis B were conducted locally employing Abbott’s Determine test kits (Abbott Laboratories, Tokyo, Japan). Reactive sera for syphilis were referred to the South Eastern Area Laboratory in Sydney, Australia for confirmatory *Treponema pallidum* particle agglutination assays and fluorescent treponema absorbance tests. In addition, frozen urine samples were referred for polymerase chain reaction testing for *Chlamydia trachomatis*, *Neisseria gonorrhoeae*, *Mycoplasma hominis* and *Ureaplasma urealyticum*.

### Data management and analysis

Data were entered into an Access database (Microsoft Corporation, Redmond, Washington, USA) on a password-protected computer and checked and validated using Epi Info, version 3.3.2 (Centers for Disease Control and Prevention, Atlanta, Georgia, USA). Descriptive statistics such as measures of central tendency were performed for sociodemographic characteristics. χ^2^ tests were used to assess associations between key determinants and chlamydia infection. The significance level was set at *P* < 0.05. All analyses were performed using SPSS, version 15.0 (SPSS Inc., Chicago, Illinois, USA).

### Ethics

At the time of this study, there was no standing human research ethics committee in Kiribati. Therefore, with the support of the Ministry of Health in Kiribati, ethics approval was granted by the Human Research Ethics Committee at the University of New South Wales, Australia (HREC 06313).

## Results

### Participant characteristics

A total of 80 women, 50 (62.5%) from South Tarawa and 30 (37.5%) from Kiritimati island, participated in this study. The mean age of participants was 21 (SD = 3.6) years with the majority aged 18–20 (*n* = 47, 58.8%). Sociodemographic characteristics of participants by location are reported in **Table 1**. Half of the study participants had been married (*n* = 40, 50%), and 32 (40%) reported ever being pregnant. Most women did not live with a partner (*n* = 64, 80%) and were away from home (left home and lived with peers due to stigma against sex work and pressure from family members) during the prior 12 months (*n* = 50, 62.5%). More than half (*n* = 45, 56.3%) of the women had never attended school, and 74 (92.5%) were unemployed (other than transactional sex) during the time of interview.

**Table 1:** Demographic characteristics of young women who boarded fishing vessels for sex work in the past 12 months, Kiribati, 2007

Characteristics	Overall (*n* = 80) *n*(%)*	South Tarawa (*n* = 50) *n*(%)	Kiritimati island (*n* = 30) *n*(%)
**Age group (years)**			
18–20	47 (58.8)	29 (58.0)	18 (60.0)
21–24	22 (27.5)	17 (34.0)	5 (16.7)
≥ 25	11 (13.8)	4 (8.0)	7 (23.3)
**Ever been married**			
Yes	40 (50.0)	27 (54.0)	13 (43.3)
No	40 (50.0)	23 (46.0)	17 (56.7)
**Ever been pregnant**			
Yes	32 (40.0)	20 (40.0)	12 (40.0)
No	48 (60.0)	30 (60.0)	18 (60.0)
**Living arrangements**			
Living with partner	10 (12.5)	7 (14.0)	3 (10.0)
Not living with partner	64 (80.0)	38 (76.0)	26 (86.7)
Not stated	6 (7.5)	5 (10.0)	1 (3.3)
Employment status (other than transactional sex)*			
Employed	6 (7.5)	5 (10.0)	1 (3.3)
Unemployed	74 (92.5)	45 (90.0)	29 (96.7)
**Education**			
Attended school	35 (43.8)	22 (44.0)	13 (43.3)
Never attended school	45 (56.3)	28 (56.0)	17 (56.7)
Away from home during last 12 months**			
Yes	50 (62.5)	34 (68.0)	16 (53.3)
No	29 (36.3)	15 (30.0)	14 (46.7)
No response/refused	1 (1.3)	1 (2.0)	0 (0)

Note: Percentages may not add to 100% due to rounding.

* Current employment status at the time of interview.

** Women left home and lived with peers due to stigma and pressure from family members.

### Behavioural characteristics

A higher proportion of women boarded fishing vessels for the purpose of having sex and drinking alcohol (*n* = 56, 70%) than having sex only (*n* = 6, 7.5%) (**Table 2**). Most women (*n* = 69, 86.3%) reported having a single partner during each visit. Although condom awareness (ascertained by the question: Have you ever heard of a male condom?) was high (90% in South Tarawa and 83.3% in Kiritimati island) (data not shown), there was infrequent use of condoms (37.5%). Only 9 (11.3%) women reported using condoms every time during sex.

**Table 2:** Behavioural characteristics of women who boarded fishing vessels for sex work in the previous 12 months, Kiribati, 2007

**-**	Overall (*n* = 80) *n*(%)*
Number of sex partner(s) during each visit	
One partner only	69 (86.3)
History of multiple sex partners during a single visit	10 (12.5)
Not stated	1 (1.3)
**Any condom use**	
Yes	30 (37.5)
No	21 (26.3)
Don’t know	2 (2.5)
No response or refused	27 (33.8)
**Frequency of condom use during the previous 30 days**	
No sex work during the previous 30 days	7 (8.8)
Every time	9 (11.3)
Sometimes	30 (37.5)
Never	4 (5.0)
Don’t know	1 (1.3)
No response or refused	28 (35.0)
Missing	1 (1.3)
**Activity on ship**	
Sex and alcohol consumption	56 (70.0)
Sex only	6 (7.5)
No response or refused	18 (22.5)

* Percentages may not add to 100% due to rounding.

### Prevalence of STIs and HIV

More than half (*n* = 46, 57.5%) of the women in our study were diagnosed with an STI. Of these, the majority (*n* = 27, 58.7%) tested positive for a single STI. Overall, the prevalence of chlamydia was 25%, syphilis was 6.3% and gonorrhoea was 2.5% (**Table 3**). Thirty-two (40%) and eight (10%) women were diagnosed with mycoplasma and ureaplasma, respectively. None of the women tested positive for HIV. Prevalence of chlamydia in women was strongly associated with younger age at first sexual intercourse (*P* = 0.02) and total number of sexual partners (*P* = 0.02) but not associated with age or level of education (**Table 4**).

**Table 3:** Frequency and prevalence of STIs and agents among young women boarding fishing vessels for sex work, Kiribati, 2007

STI	Frequency	Prevalence (%)
Chlamydia	20	25.0
Gonorrhoea	2	2.5
Syphilis	5	6.3
HIV	0	0
Mycoplasma	32	40.0
Ureaplasma	8	10.0
No STI detected*	34	42.5
Any STI	46	57.5
1 STI	27	33.8
2 STIs	17	21.3
3 STIs	2	2.5

HIV = human immunodeficiency virus; STI = sexually transmitted infection.

* Refers only to STIs and agents tested in this study, namely chlamydia, gonorrhoea, syphilis, HIV, mycoplasma and ureaplasma.

**Table 4:** Frequency of genital *Chlamydia trachomatis* infections by selected demographic and risk factors among young women boarding fishing vessels for transactional sex, Kiribati, 2007

Characteristic	Overall (*n* = 80) *n*(%)	Presence of *Chlamydia* infection (*n* = 20) *n*(%)*	*p* value
Current age			
< 21 years	47 (58.8)	9 (19.1)	0.15
≥ 21 years	33 (41.3)	11 (33.3)	
**Age of first sexual intercourse**			
< 18 years	33 (41.3)	5 (15.2)	0.02
≥ 18 years	35 (43.8)	14 (40.0)	
Not stated	12 (15.0)	1 (12.5)	
**Education status**			
Never attended school	45 (56.3)	10 (22.2)	0.52
Attended school	35 (43.8)	10 (28.6)	
**Number of sexual partners during last 7 days**			
1 partner	23 (28.8)	4 (17.4)	0.02
≥ 2 partners	31 (38.8)	13 (41.9)	
Not stated	26 (32.5)	3 (11.5)	

* Percentages were calculated by dividing the number of women in the presence of chlamydia infection column by the corresponding women in the overall column. For instance, of the 47 women younger than 21 years old, 9 or 19.1% of them had chlamydia.

## Discussion

The results of this study show that women who board foreign fishing vessels for sex work tend to have limited education and are young, are not living with a partner and are otherwise unemployed. This study assesses the prevalence of risk behaviours and select STIs among women boarding foreign fishing vessels for transactional sex in Kiribati and shows that more than half (57.5%) of the women were diagnosed with an STI.

The prevalence of STIs in our study was twice as high as the overall prevalence among sex workers in Papua New Guinea (28.7). (*7*) In addition, the prevalence of chlamydia in our study population was higher than other studies of female sex workers. (*7*, *12*, *13*) The 25% chlamydia incidence in the women in our study is nearly twice that shown in a study of pregnant women from the general population in Kiribati. (*9*) This substantial difference highlights the increased STI risk for sex workers and emphasizes the need for public health interventions. In our study, 40% of the participants had ever been pregnant, which underscores the need for prenatal STI screening in this population so any treatable infections can be treated as early as possible. Consistent with studies from other nations, (*14*, *15*) our results also revealed a significant association between chlamydia and a history of contact with two or more sexual partners.

Consistent with previous findings among sex workers in China, (*16*) the prevalence of urogenital *Mycoplasma hominis* (40%) and *Ureaplasma urealyticum* (10%) was high in this study population. A high prevalence of *Mycoplasma spp.* and *Ureaplasma spp.* could indicate a loss or decrease of lactobacillus species from the vaginal flora and is associated with bacterial vaginosis. Previous studies have shown that the loss of hydrogen peroxide (H_2_O_2_)-producing *Lactobacillus spp*. can lead to the overgrowth of pathogenic organisms and can increase the risk of transmission of STIs and HIV. (*17*)

None of the women in our study tested positive for HIV; however, high rates of chlamydial infection, which is associated with an increased risk of HIV transmission (*18*) and low rates of consistent condom use, (*19*) could increase transmission risk if HIV were introduced into this population.

The high prevalence of STIs among this population of women flags the susceptibility of this group to a range of related severe and permanent health conditions, including but not limited to infertility, ectopic pregnancy, pelvic inflammatory disease and human papillomavirus-associated cancers. (*20*, *21*) In addition, a high prevalence of STIs in the sex worker population increases the risk of introducing STIs into the general population, especially given the low adherence to safer sex practices by sex workers. (*22*)

Participants had a high awareness of condom use (90% and 83.3% for South Tarawa and Kiritimati island, respectively), but many reported minimal or inconsistent condom use. A possible explanation for the infrequent use of condoms could be the lack of availability of condoms when needed. Future studies should investigate the reasons for the inconsistency in condom use contrasted with the high awareness reported. Even among the women reporting condom use, a high percentage of them had an STI (data not shown). This could indicate response bias with participants reporting higher condom use to meet the perceived expectations of the interviewers. Another possible explanation could be that condoms were used incorrectly, for example, the lack of condom use during sexual foreplay.

In our study, a high proportion of women (56%) had never attended a school. Of those that attended a school, high dropout rates were reported from secondary schools. Low education level is not only associated with entry into sex work (*23*) but is also a risk factor for STIs among women engaged in sex work. (*24*) Besides transactional sex, most women (92.5%) in our study were unemployed. Fewer employment opportunities in Kiribati coupled with a lack of formal education makes it challenging for these women to secure jobs. These socioeconomic factors are some of the drivers for women to enter sex work in Kiribati.

This study has some limitations that merit consideration. First, the small sample size could have reduced the power of the study to detect true sociodemographic and behavioural differences between those with chlamydia and those without. Recruiting participants was challenging due to Kiribati cultural norms that disapprove of engaging in sex for money and dictate that unmarried women should not be sexually active. Nonetheless, the sample size of our study reflects the small size of the population of women boarding foreign fishing vessels. Second, the survey responses may reflect socially desirable responses; however, we aimed to minimize this bias by using trained counsellors who assured the participants that their responses would be private, confidential and secure. In addition, the counsellors were trained and advised to adopt a non-judgmental approach to the participants’ responses. Third, behavioural characteristics, such as condom use, were self-reported and may have been subject to recall issues. Fourth, due to the cross-sectional nature of our study, associations between various factors and STIs cannot be interpreted to infer causality.

Despite these limitations, this is the first study to be conducted among this marginalized group of women in Kiribati. The study has identified risk factors for STI transmission and barriers that need to be addressed specific to this high-risk population. Economic drivers such as limited employment opportunities coupled with the lack of education are some of the reasons women board foreign fishing vessels for sex work. (*25*)

The high prevalence of STIs among the women in our study warrants an immediate response to prevent wider community transmission. Public health interventions such as periodic presumptive treatment to decrease morbidity from STIs and shorten the duration of infectiousness to prevent further spread into the community must be implemented promptly. (*26*) We recommend a combined approach, including enhancing existing communicable disease surveillance systems nationwide, strengthening health education to women at high risk of STIs and to the general population, tailoring health programmes to make them more acceptable and accessible to women engaged in transactional sex and promoting efforts to destigmatize these women in Kiribati society. As the prevalence of STIs in this key population group is high, we advocate for follow-up studies to assess the trends of STIs in this population. Future studies could also assess any impact this population may have on the overall epidemiology of STIs or HIV in Kiribati.
